# Improving Child Oral Health: Cost Analysis of a National Nursery Toothbrushing Programme

**DOI:** 10.1371/journal.pone.0136211

**Published:** 2015-08-25

**Authors:** Yulia Anopa, Alex D. McMahon, David I. Conway, Graham E. Ball, Emma McIntosh, Lorna M. D. Macpherson

**Affiliations:** 1 Dental School, College of Medical, Veterinary and Life Sciences, University of Glasgow, Glasgow, United Kingdom; 2 Health Economics and Health Technology Assessment, College of Medical, Veterinary and Life Sciences, University of Glasgow, Glasgow, United Kingdom; 3 NHS Fife, Leven, United Kingdom; University of Washington, UNITED STATES

## Abstract

Dental caries is one of the most common diseases of childhood. **The aim** of this study was to compare the cost of providing the Scotland-wide nursery toothbrushing programme with associated National Health Service (NHS) cost savings from improvements in the dental health of five-year-old children: through avoided dental extractions, fillings and potential treatments for decay.

**Methods:**

Estimated costs of the nursery toothbrushing programme in 2011/12 were requested from all Scottish Health Boards. Unit costs of a filled, extracted and decayed primary tooth were calculated using verifiable sources of information. Total costs associated with dental treatments were estimated for the period from 1999/00 to 2009/10. These costs were based on the unit costs above and using the data of the National Dental Inspection Programme and then extrapolated to the population level. Expected cost savings were calculated for each of the subsequent years in comparison with the 2001/02 dental treatment costs. Population standardised analysis of hypothetical cohorts of 1000 children per deprivation category was performed.

**Results:**

The estimated cost of the nursery toothbrushing programme in Scotland was £1,762,621 per year. The estimated cost of dental treatments in the baseline year 2001/02 was £8,766,297, while in 2009/10 it was £4,035,200. In 2002/03 the costs of dental treatments increased by £213,380 (2.4%). In the following years the costs decreased dramatically with the estimated annual savings ranging from £1,217,255 in 2003/04 (13.9% of costs in 2001/02) to £4,731,097 in 2009/10 (54.0%). Population standardised analysis by deprivation groups showed that the largest decrease in modelled costs was for the most deprived cohort of children.

**Conclusions:**

The NHS costs associated with the dental treatments for five-year-old children decreased over time. In the eighth year of the toothbrushing programme the expected savings were more than two and a half times the costs of the programme implementation.

## Introduction

Oral health is an integral part of general health and is essential for the well-being of individuals. The oral cavity contributes to health-related quality of life at the biologic and psychosocial levels [1]. Dental caries is one of the most common diseases of childhood [2], impacting quality of life through pain, infection, diet, and loss of sleep. Caries can also lead to time lost from school for children and time off work for parents/carers [3]. Caries can be effectively prevented and controlled [2, 4] substantially improving quality of life and child morbidity [5–7]. The Scottish Government and National Health Service (NHS) Scotland are at the forefront of child oral health improvement and they have been funding a nation-wide supervised nursery toothbrushing program since 2001 and since 2006, the *Childsmile* program, which incorporated the toothbrushing program [8, 9]. The overarching aim of these programmes is to reduce childhood caries and narrow health inequalities.

Our previous study has shown that the major improvement in the dental health and the reduction in dental health inequalities among five-year-old children in Scotland observed over the past decade was associated with the introduction and uptake of the nursery toothbrushing program. Child oral health has improved in the face of flat-lining trends in general child health indicators [10].

The Christie Commission Report is a key driver for public sector policy and budget allocation in Scotland—with an emphasis on addressing inequalities and the focus of what it defined as a “preventative spend” approach. This approach aims to shift both action and resource into preventative action with the ultimate aim of improving health outcomes and saving resources long term [11].

Dental programmes in general, and oral health preventative programmes in populations in particular, rarely receive the same level of attention as medical care among policy makers with regard to the cost-effective allocation of scarce health care resources [12]. Policymakers may consider oral health to be less important than other health needs [13]. As a result, the application of economic evaluation in preventive dentistry [14] and dental public health [15] remains limited. Our literature search identified only two economic evaluations conducted in relation to fluoridated toothpaste programmes [16, 17].

The aim of this study was to compare the cost of providing the Scotland-wide nursery toothbrushing program with the associated NHS expected cost savings from an improvement in dental health of five-year-old children. The expected cost savings were estimated as a proxy for the opportunity cost (benefits forgone) of the freed-up dental resources and were calculated as a direct function of the averted decayed, missing and filled teeth in five-year-olds. Without information on preference-based quality of life estimates such as the quality adjusted life year for caries-related dental health states and little indication in the literature relating to the economic value of the improved dental health via reduction in dental caries, the aim of this cost analysis is to provide initial estimates of cost savings through improved dental health outcomes (decrease in the number of decayed, missing and filled teeth in 5-year-old population). It is important to note that such expected cost savings provide an estimate for dental health resources now released and re-deployed and not financial resources to be accrued on a balance sheet.

## Methods

### Study design

This cost analysis adopts a health service perspective and combines the total costs of the nursery toothbrushing program in each of the Scottish Health Boards in the 2011/12 financial year with the averted total costs of actual and anticipated dental treatments caused by dental caries in 5-year-old children. In this research, ‘actual’ treatments are defined as the dental treatments, delivered every year (such as tooth fillings and extractions) and recorded within various dental data collection systems, whereas ‘anticipated‘ relate to treatments assumed to happen in the coming years taking in account possible management options for decayed teeth, i.e. they may be filled, extracted or left untreated until exfoliation. In the case of ‘anticipated treatments’ the probabilities of different fates of decayed primary teeth have been incorporated into the cost analysis.

Currently there are 14 territorial Health Boards in Scotland, with the total population of each board ranging from around 21,000 to 1.2 million people, as of 2012 mid-year estimates [18]. In Scotland the costs for dental treatment in 0–17 years old children/adolescences are met by the Government through NHS payments [19]. The total costs of actual and anticipated dental treatments were estimated by combining the data on the numbers of decayed, missing and filled teeth per child from the national multiple cross-sectional dental epidemiology inspections of 5-year-old children in Scotland between 1999/00 and 2009/10 with the unit costs of treatment for decayed, filled and missing teeth. These costs were then extrapolated to the population level, using the population estimates of 5-year-olds for each year of the analysis, in order to reflect the total budgetary impact. As there were no national dental inspections of five year old children conducted in 2000/01, 2001/02, 2004/05, 2006/07 and 2008/09, the dental treatment costs for these years were linearly interpolated based on the costs estimated for the years with the dental inspection results available. The interpolation was performed by each cost component: costs of decayed, missing and filled teeth.

Unit costs of actual and anticipated dental treatments resulting from caries in five-year-old children were calculated in 2009 British pound sterling (GBP, £). Where cost data were not available for the 2009/10 financial year, the available costs for more recent years were deflated using 3% per year deflation rate [20–22]. All costs were reported in the base year 2009/10. Discounting was not employed as costs and savings were estimated independently within each year and compared using a common base year to reflect the total impact on resources.

Despite the fact that this study is a partial economic evaluation, the authors followed the Consolidated Health Economic Evaluation Reporting Standards (CHEERS) guidelines (http://www.equator-network.org/reporting-guidelines/cheers/) for reporting economic evaluations of health interventions [23].

### Data

#### Implementation costs: nursery toothbrushing program costs

In Scotland there is funding for all children to attend nursery establishments at the ages of three and four years, and a very high proportion of children attend [24]. Every three- and four-year-old child attending a nursery (full- or part-time) participating in the national toothbrushing program is offered free, daily, supervised toothbrushing within the nursery establishment. A small pea-sized amount of toothpaste containing at least 1000 ppm fluoride is used. The length of brushing is at least 2 min. Toothbrushes and brushing techniques are appropriate to the age and ability of the child and children are closely supervised when brushing their teeth [10]. The vast majority of nurseries participate in the toothbrushing programme: by 2005/06 the average nursery participation rate had reached around 80%, further increasing to 95% in 2006/07, and staying at or above this level onwards.

Data on the total costs of implementing the nursery toothbrushing program in the 2011/12 financial year were requested from each of the 14 Scottish Health Boards and then deflated to 2009/10 at 3% per year rate. The assumption was that the annual costs of nursery toothbrushing remained constant over time, so the value for 2009/10 was applied retrospectively to all previous years. The annual implementation costs of the nursery toothbrushing program included: staff salaries, transport and travel, administration/office costs, staff training and toothbrushing resources costs (toothbrushes, toothpaste, toothbrushing packs for home use, storage units for toothbrushes, demonstration aids, and other consumables).

#### Dental inspection data

In order to quantify the averted costs due to reduced dental caries rates, changes in the numbers of decayed, missing and filled teeth per 5-year-old child for the duration of the study were identified, measured, valued and compared with the pre-toothbrushing *status quo*. We obtained the data on the numbers of decayed, missing and filled teeth per child from the national dental inspections of 5-year-olds [Scottish Health Boards’ Dental Epidemiological Program (1999/2000); National Dental Inspection Program (2002/03-2009/10)–full reports are available online [25, 26]]. The national dental inspections of five year old children were conducted on average every second year and covered each of the Health Boards. Data from all 14 Boards were analysed in this study. The dental data were generated as part of the national oral health monitoring system for school children and no further ethical approval was required for the analysis of these data. There is an option to opt-out of this program but opt-in consent is not applicable. Each child’s home postcode had been assigned a Carstairs socio-economic deprivation score—Depcat [27]–with Depcat 1 being the most affluent, and Depcat 7 being the most deprived. The Carstairs score for each postcode sector is not a measure of the extent of material well-being or relative disadvantage experienced by individuals, but is rather a summary measure applied to populations contained within small geographic localities. The Carstairs score is the accepted socio-economic measure available in Scotland for historic analysis. A total of 62,419 anonymised child dental records were analysed with the numbers of the five-year-old children inspected, and proportions of the relevant Scottish population, ranging from 6,766 (11.0%) to 12,067 (23.2%) in various inspection years.

### Time period

The time period for the cost analysis was 1999/00 to 2009/10. The analysis start year of 1999/00 was chosen to include the dental inspection prior to the Scotland-wide roll-out of the nursery toothbrushing program in 2001/02, while the end (2009/10) was chosen to coincide with the analysis period end used in our previous paper on nursery toothbrushing [10].

### Costing methods

#### Unit costs of actual and anticipated dental treatments

Individual unit costs of the treatments for filled, missing and decayed teeth were based on treatment probabilities, resource use for treatments and available dental remuneration data as outlined below:

*The cost of a filling* was derived from the Scottish Statement of Dental Remuneration (SDR), Amendment No 115 [28], used in combination with the Information Services Division (ISD) of the NHS data on dental treatment claims and costs for 2009/10 financial year [29]. The SDR costs of the four available restorative treatments (based on clinical evidence): filling, preformed metal cap, vital and non-vital pulpotomy were weighted according to the relative probability of each type of treatment performed by NHS General Dental Practitioners in Scotland in 2009/10 (Table 1). The SDR codes and treatments related to primary teeth in patients under 18 years of age. Further breakdown by age of a patient was not available. Our assumption was that fillings in primary teeth would not fail, and hence no repeated treatments would be required.

**Table 1:** Unit cost of a filling, 2009 GBP.

Description	Child number of claims	SDR codes	SDR unit cost	Weighting	Weighted cost
Filling	78,039	4401	£7.87	89.3%	£7.03
Filling (occasional treatment)	625	6001	£13.98	0.7%	£0.10
Preformed metal cap	4,005	4402 and 6002	£20.69	4.6%	£0.95
Vital pulpotomy	933	4403 and 6003	£8.27	1.1%	£0.09
Non vital pulpotomy	3,744	4404 and 6004	£15.73	4.3%	£0.67
Total	87,346			100.0%	
**Expected cost of a filling**					**£8.84**

*The costs of dental extractions* were calculated as a function of the number of teeth extracted in one session. It was assumed that for zero to five year old children tooth extractions were performed under a local anaesthetic (LA) if only one tooth was to be extracted, and general anaesthesia (GA) was used if two or more teeth were to be extracted. The data supplied on request by ISD showed that in 2009/10 the average number of teeth extracted per visit under LA for 0-5-year-old patients was 1.24 [30], which supports our assumption that approximately only one tooth per child was extracted under LA. The expected cost of an extracted tooth was based on the costs of tooth extractions using both LA and GA. It was assumed that the approximate proportion of the number of extractions under LA to those performed under GA in 4-7-year-old children was 55%: 45%, based on the data on dental LA claims by single year of child age received on request from ISD [30] and data on the numbers of dental GA episodes in children in 2009/10 [31] by single year of age, also received on request from ISD. The age group of 4–7 years was chosen as the children examined in the dental inspections were on average 5.5 years old, while the range of ages across Scotland was 4–7 years (NDIP). Moreover, a paper by Levine et al. (2002) [32] indicated that the majority of carious lesions in primary teeth presented by the age of 6 years, and carious teeth had a mean survival time in the range of 649–766 days before being filled or extracted (unless they were left untreated until exfoliation). By applying the above weightings the weighted LA and GA costs were calculated first, and then the overall expected cost of an extracted tooth (Table 2), which was then used in calculation of the cost of a decayed tooth (Table 3). The cost of a tooth extracted under LA was based on Amendment No 115 of SDR [28] and the ISD data on dental treatment claims and costs for 2009/10 [29]. The cost of teeth extracted under GA was based on the 2011/12 financial year median gross cost per dental daycase by hospital taken from the ISD Scottish Health Service Costs (known as the Costs Book) data on specialty costs and activity [33], which was then deflated to 2009/10 at 3% per year rate (Table 2).

**Table 2:** Unit cost of a tooth extraction, 2009 GBP.

**a) Cost of a tooth extracted under local anaesthetic (LA)**			
**Description**		**SDR code**	**SDR unit cost**
Extraction of teeth (fee per course of treatment)		2101	£7.87 (extraction of 1 tooth)
Additional fee for each visit for extraction, including the first		2121	£6.46
**Cost of a tooth extracted under LA**			**£14.33**
Assumption: LA was used if a child had one tooth missing / extracted (based on the national dental inspection results).			
**b) Cost of a dental general anaesthesia procedure for multiple teeth extraction (GA)**			
£694.29 in 2011/12; deflated at the rate of 3% per year to 2009/10, this equals **£653.26**. Assumption: GA was used if a child had two or more teeth missing / extracted (based on the national dental inspection results).			
**c) Calculations of expected cost of an extracted tooth**			
Assumption: Ratio of the number of extractions performed using LA to those performed under GA in 4-7-year-old children was 55%: 45%			
**Description**	**Cost**	**Weighting**	**Weighted cost**
Dental extractions under LA	£14.33	55%	£7.93
Dental extractions under GA	£653.26	45%	£291.59
**Expected cost of an extracted tooth**			**£299.53**
(This cost was later used in the calculation of the cost of a decayed tooth.)			

**Table 3:** Unit cost of a decayed primary tooth, 2009 GBP.

Description	Values / Calculations	Sources
Total No. of primary teeth with decay (followed up until exfoliation or extraction):	1587 (100.0%)	Levine RS, Pitts NB, Nugent ZJ (2002). The fate of 1,587 unrestored carious primary teeth: a retrospective general dental practice based study from northern England. British dental journal 193(2):99–103.
No. of teeth extracted due to pain:	190 (12.0%)	
No. of teeth without pain extracted (under GA, together with the painful teeth):	178 (11.2%)	
No. of teeth filled:	60 (3.8%)	
We re-calculated the number of filled teeth, assuming that some of the non-painful teeth extracted under GA might have been filled instead:	a) The likelihood of a primary decayed tooth being filled = No. of teeth filled / Total No. of primary teeth with decay = 60 / 1587 = 0.038	Calculations by the authors using the teeth numbers from Levine et al. (2002)
	b) The “additional” number of primary decayed teeth which might have been filled instead of being extracted (no pain) = Number of carious teeth without pain that were extracted under GA * The likelihood of a tooth being filled = 178 * 0.038 = 7 teeth	
Number of filled teeth used in this analysis:	60 + 7 = 67	
Total No. of teeth “treated” (filled or extracted)	= extracted teeth (due to pain) + filled teeth = 190 + 67 = 257	
Likelihood of a decayed tooth being “treated” (filled or extracted; in contrast with those decayed teeth that are left untreated until exfoliation)	= Total No. of teeth “treated” (filled or extracted) / Total No. of primary teeth with decay = 257 / 1587 = 0.162	
Likelihood of a decayed tooth being extracted (not including decayed teeth that are left untreated until exfoliation)	= No. of teeth extracted due to pain / Total No. of teeth “treated” = 190 / 257 = 0.74	
Likelihood of a decayed tooth being filled (not including decayed teeth that are left untreated until exfoliation)	= No. of teeth filled / Total No. of teeth “treated” = 67 / 257 = 0.26	
Expected cost of extraction	£299.53	Unit cost calculated by the authors (Table 2, c)
Cost of a filling	£8.84	Unit cost calculated by the authors (Table 1)
Expected cost of a “treated” tooth (filled or extracted)	= (Cost of extraction * Likelihood of a decayed tooth being extracted) + (Cost of a filling * Likelihood of a decayed tooth being filled) = £299.53 * 0.74 + £8.84 * 0.26 = £223.95	
**Expected cost of a decayed tooth**	= Expected cost of a treated tooth (filled or extracted) * Likelihood of being “treated” = £223.95 * 0.162 = **£36.28**	

*The cost of dental decay* was calculated based on the numbers of decayed primary teeth with various fates, e.g. extracted due to pain, extracted without pain or filled, followed up in a longitudinal study [32], the likelihood of certain fates, and the unit costs of an extracted tooth and filled tooth (Table 3).

#### Expected cost savings

Expected cost savings (through averted costs of dental extractions, fillings and potential treatments for decay) were calculated for each of the subsequent years in comparison with the dental treatment costs in the base year 2001/02.

#### Deprivation

The impact of the deprivation categories sub-populations sizes was controlled for using population standardised analysis. Costs were calculated for a hypothetical cohort of 1000 children representing each Depcat based on the dental inspection results of 1999/00, the inspection prior to the national roll-out of the toothbrushing in nurseries, and 2009/10, the final year of the analysis presented in this paper. For each year and each Depcat category, the proportion of the sample that had untreated decay (by the number of decayed teeth per child), fillings (by the number of filled teeth) or missing teeth (by whether it was one missing tooth or two or more missing teeth per child) were calculated, based on the results of the national dental inspections. These proportions were then applied to a hypothetical sample of 1000 children representing each Depcat. The products were multiplied by the appropriate unit cost and then the results for the sub-cohorts by the number of teeth treated were summed to get costs of decayed, missing and filled teeth by Depcat, as below:

Cost of **Decayed Teeth** per 1000 population by Depcat = Σ (% Depcat Sample by number of teeth decayed * Cost of Decayed tooth * Number of decayed teeth * 1000)

Cost of **Filled Teeth** per 1000 population by Depcat = Σ (% Depcat Sample by number of teeth filled * Cost of Filled tooth * Number of filled teeth * 1000)

Cost of **Extracted Teeth** per 1000 population by Depcat = (% Depcat Sample with one tooth missing * Cost of LA extraction * 1000) + (% Depcat Sample with 2 or more teeth missing * Cost of GA extraction * 1000)

#### Sensitivity analysis

The estimated total costs of actual and anticipated dental treatments over time were subjected to one- and two-way sensitivity analyses (Table 4). Four scenarios were used: (i) low GA cost; (ii) high GA cost; (iii) low filling cost; and (iv) a different ratio of filled to extracted ‘treated’ decayed teeth (in contrast with decayed teeth that were left untreated until exfoliation). The low GA cost was based on the 5^th^ percentile and the high GA cost on the 95^th^ percentile of the gross cost per dental daycase by hospital in 2011/12 deflated to 2009/10 [33]. Low filling cost was based on a scenario when there were no preformed metal caps available (e.g. historically) and when potential treatments included only fillings, vital and non-vital pulpotomy. A different ratio of filled to extracted ‘treated’ decayed teeth was used as at present there is no published research that would show what proportion of decayed primary teeth were filled or extracted at a population level. As the cost of GA also influenced the expected cost of a decayed tooth, the high and low GA cost scenarios were, in fact, two-way sensitivity analyses.

**Table 4:** Sensitivity analysis scenarios, 2009 GBP (in bold italic are values that differ from the baseline scenario).

Costs / Assumptions	Scenario				
	Baseline	Low GA cost	High GA cost	Low filling cost	Different filled / extracted ‘treated’ teeth ratio
Filled tooth	£8.84	£8.84	£8.84	***£8*.*25*** *	£8.84
Decayed tooth	£36.28	***£22*.*37*** *	***£75*.*92*** *	£36.28	***£29*.*69*** *
Tooth extraction under local anaesthetic (LA)	£14.33				
Tooth extraction under general anaesthetic (GA)	£653.26	***£393*.*22*** *	***£1*, *393*.*89*** *	£653.26	£653.26
Ratio of filled to extracted ‘treated’ decayed teeth	26% / 74%				***40% / 60%*** *

* In bold italic are the values that differ from the baseline scenario.

## Results

### Nursery toothbrushing program costs

As the organisation and delivery of the nursery toothbrushing program differed across Scotland, the elements constituting the total costs provided varied from area to area. The estimated cost of the nursery toothbrushing program in Scotland was £1,762,621per year (after deflation to the 2009/10 level). For costs by Health Board see Table 5. The largest cost component was staff salary, this ranged from 49.1% to 90.4% of the total annual nursery toothbrushing cost by Health Board. Toothbrushing resources accounted for 4.4%-49.4% of the total costs. Transport and travel costs accounted for 2.7%-17.4%, administration/office costs for 0.2%-12.4%, and staff training for 0.5%-3.5% of the total nursery toothbrushing program cost per Health Board.

**Table 5:** Annual cost of nursery toothbrushing programme in 2011/12 financial year and deflated to 2009/10, by Scottish Health Board.

Mid-2011 population of all ages in each Health Board *	Mid-2011 population of 3-4-year-olds in each Health Board *	Health Board code	Cost of nursery toothbrushing programme in 2011/12	Cost of nursery toothbrushing programme deflated to 2009/10 **
Under 28,000	Under 600	1	£19,881	£18,706
		2	£30,011	£28,237
		3	£31,752	£29,875
100,000–450,000	2,000–9,000	4	£31,932	£30,045
		5	£38,166	£35,910
		6	£41,533	£39,078
		7	£84,505	£79,511
		8	£99,318	£93,448
		9	£101,180	£95,200
		10	£140,125	£131,844
Over 550,000	Over 12,000	11	£218,493	£205,580
		12	£294,944	£277,513
		13	£341,845	£321,642
		14	£399,650	£376,031
**5,299,900**	**116,160**	**Total**	**£1,873,335**	**£1,762,621**

* Source: National Records of Scotland (2012), Estimated population by sex, single year of age and administrative area, mid-2011 (http://www.nrscotland.gov.uk/files/statistics/population-estimates/mid-2011/11mype-cahb-table2.xls); accessed 29 May 2015)

** At 3% per year deflation rate.

### Unit costs of filled, decayed and missing teeth

Table 4, ‘baseline scenario’, shows the estimated unit cost of a filled tooth of £8.84, the cost of a tooth extracted under LA £14.33, the estimated cost of a GA procedure for multiple teeth extraction (£653.26) and the expected unit cost of a decayed tooth (£36.28).

### Numbers of filled, decayed and missing teeth

The results of the extrapolation of the numbers of filled and decayed teeth, as well as the numbers of five year old children with one missing tooth (due to decay) and two or more missing teeth to the Scottish five year old population level are shown in Table 6. The teeth/children numbers were extrapolated from the results of the national dental inspections to the level of the five year old population within each year of the analysis. During the 1999/00-2009/10 period the numbers of decayed teeth in the population and numbers of children with missing teeth decreased dramatically. The number of decayed teeth decreased from 107,925 to 57,167 (a 47.0% decrease in comparison with the 1999/00 figure). The number of children with one missing tooth decreased from 1,615 to 776 (52.0% decrease), while the number of children with two or more missing teeth decreased from 6,479 to 2,837 (56.2% decrease). The number of filled teeth in the five year old population decreased from 19,030 to 10,909, which is a 42.7% decrease in comparison with 1999/00.

**Table 6:** Estimated number of filled and decayed teeth and number of children with missing teeth in five year old population in Scotland, 1999/00–2009/10.

Year	Number of filled teeth in 5 y.o. population	Number of decayed teeth in 5 y.o. population	Number of children with missing teeth in 5 y.o. population	
			1 tooth missing	2 or more teeth missing
**99/00**	19,030	107,925	1,615	6,479
**00/01** *				
**01/02** *				
**02/03**	17,857	113,844	1,937	7,139
**03/04**	15,849	97,010	1,504	5,921
**04/05** *				
**05/06**	12,966	76,545	1,062	4,560
**06/07** *				
**07/08**	11,777	63,555	1,122	4,084
**08/09** *				
**09/10**	10,909	57,167	776	2,837

* No national dental inspections of 5 year old children were conducted in 2000/01, 2001/02, 2004/05, 2006/07 and 2008/09. Dental treatment costs for these years were linearly interpolated based on the costs for the years with the dental inspection results available.

### Expected cost savings

**Fig 1:**
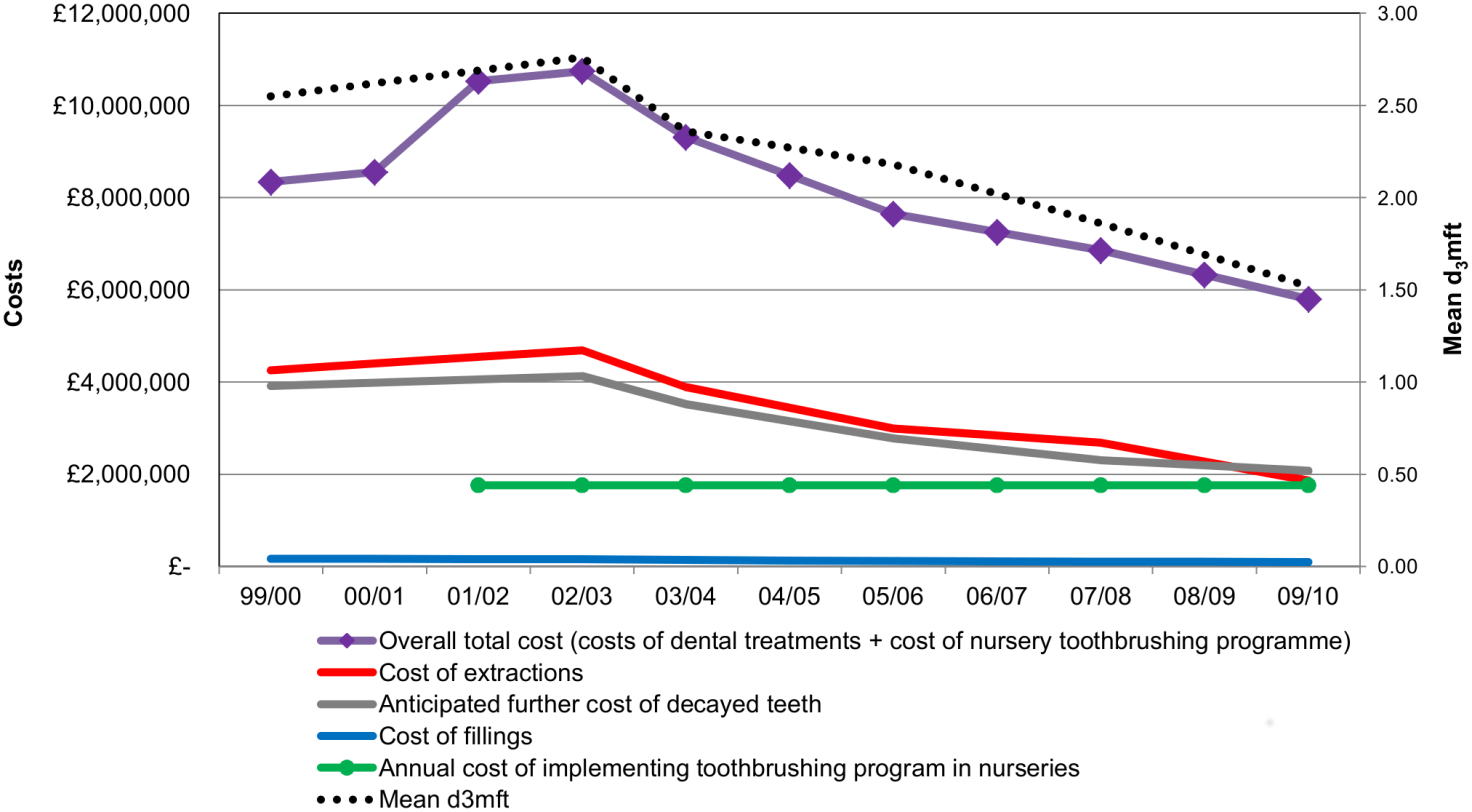
Costs of actual and anticipated dental treatments in five-year-old children (baseline scenario), cost of nursery toothbrushing programme and d_3_mft over time—Scotland, by financial year. d_3_mft index is the number of obviously decayed, missing (due to decay) and filled teeth per child. The “3” in the d_3_mft index indicates decay into dentine.

Fig 1 shows the costs of actual and anticipated dental treatments in five-year-old children by component and implementation cost of the nursery toothbrushing programme. It also shows mean d_3_mft index, a common dental metric, which is the number of obviously decayed, missing (due to decay) and filled teeth per child. The “3” in the d_3_mft index indicates decay into dentine. The declining trend of dental treatments costs is clearly evident in Fig 1. As discussed in our previous paper, it is a direct function of the improved dental health of children that is associated with the nursery toothbrushing program [10]. Among the three dental treatment cost components the lowest annual costs were associated with fillings, whereas the highest annual costs were associated with extracted teeth. The estimated total cost of actual and anticipated dental treatments, which is the sum of costs of decayed, extracted and filled teeth, in the baseline year 2001/02 was £8,766,297, while in 2009/10 it was £4,035,200. Fig 1 also shows the overall total dental care costs, which is the sum of all dental treatment costs and the cost of the nursery toothbrushing programme.

**Fig 2:**
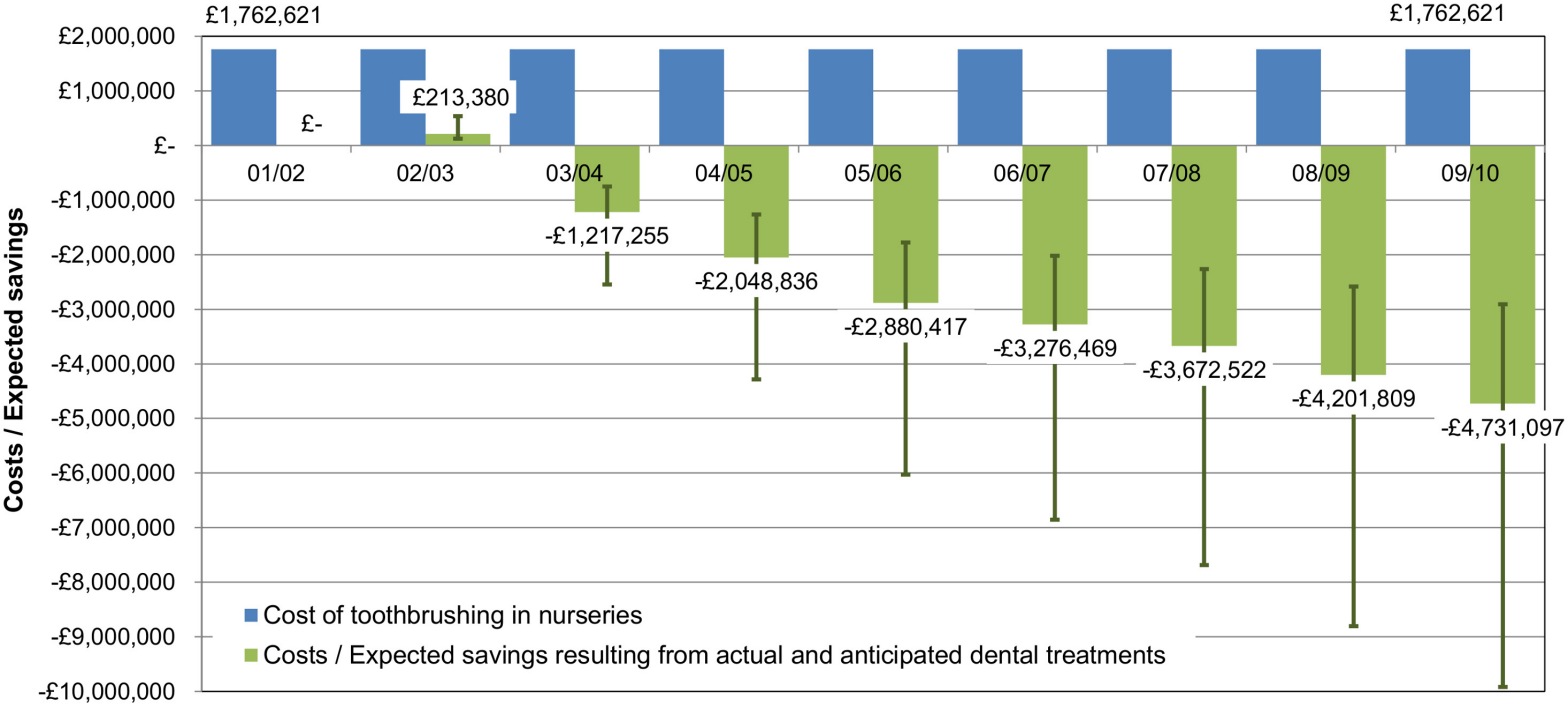
Annual cost of nursery toothbrushing programme and costs / expected savings resulting from actual and anticipated dental treatments—in comparison with 2001/02 dental treatment costs. The figure shows data for Scotland, 2001/02-2009/10 financial year. The whiskers represent costs / expected savings resulting from actual and anticipated dental treatments in the case of a ‘low GA cost’ and ‘high GA cost’ scenarios.

The expected savings resulting from actual and anticipated dental treatments are shown in Fig 2. In 2002/03 the costs of actual and anticipated dental treatments increased by £213,380 (2.4%) in comparison with the dental treatment costs in 2001/02. However, in the following years the costs decreased dramatically with the estimated savings ranging from £1,217,255 (13.9%) in 2003/04 to £4,731,097 (54.0%) in 2009/10 in comparison with the baseline. Within three years from the national roll-out of the nursery toothbrushing program the expected cost savings (freed-up resources) outweighed the costs of implementing the toothbrushing program and by eight years the expected cost savings were over two and a half times these costs.

### Costs and expected cost savings per child

Table 7 shows the cost of actual and anticipated dental treatments per 5 year old child, expected savings per 5 year old child, and cost of the nursery toothbrushing programme per 3–4 year old child participating in toothbrushing. The highest decrease in costs per child was observed for the cost of dental extractions as well as for anticipated further cost of decayed teeth. The decline in cost of fillings per child was less prominent over time. The expected savings resulting from actual and anticipated dental treatments per 5 year old child ranged from £21.43 in the second year of the toothbrushing programme, 2003/04, to £86.31 in the eighth year, 2009/10. There was little variation in the average cost per 3–4 year old child in the Scottish population over time, due to one of our main assumptions that the cost of the toothbrushing programme was constant over time. The range of the cost per 3–4 year old child in the population was from £15.26 to £16.89 in various years.

**Table 7:** Population of 5 year old / 3–4 year old children, costs and expected savings of dental treatments per 5 year old child, and cost of nursery toothbrushing programme per 3–4 year old child, Scotland, 1999/00–2009/10 financial years.

Year	Population of 5 y.o. children in Scotland *	Total cost of actual and anticipated dental treatments (per 5 y.o. child), £	Cost of dental extractions (per 5 y.o. child), £	Anticipated further cost of decayed teeth (per 5 y.o. child), £	Cost of fillings (per 5 year old child), £	Costs / Expected savings resulting from actual and anticipated dental treatments (per 5 y.o. child), £ **	Population of 3–4 y.o. children in Scotland *	Cost of nursery toothbrushing programme (per 3–4 y.o. child in population), £ ***
**99/00**	61,399	135.83	69.31	63.77	2.74			
**00/01**	59,175	144.54	74.37	67.38	2.78			
**01/02**	58,088	150.91	78.27	69.87	2.78	*Comparator year*	115,506	15.26
**02/03**	58,624	153.17	80.03	70.45	2.69	3.64	112,387	15.68
**03/04**	56,803	132.90	68.47	61.96	2.47	-21.43	108,812	16.20
**04/05**	55,929	120.11	61.54	56.29	2.28	-36.63	105,891	16.65
**05/06**	53,553	109.91	55.91	51.86	2.14	-53.79	104,355	16.89
**06/07**	52,843	103.89	53.73	48.09	2.07	-62.00	104,464	16.87
**07/08**	52,093	97.78	51.52	44.26	2.00	-70.50	107,394	16.41
**08/09**	53,135	85.90	42.80	41.21	1.89	-79.08	110,095	16.01
**09/10**	54,812	73.62	34.02	37.84	1.76	-86.31	111,688	15.78

All costs / expected savings are reported in 2009 British pound sterling (GBP, £).

* Source: General Register Office for Scotland / National Records of Scotland (2000–2010), Mid-year estimated population by sex, single year of age and administrative area– 1999–2009 years. Based on the 2001 Scotland’s Census (http://www.nrscotland.gov.uk/statistics-and-data/statistics/statistics-by-theme/population/population-estimates/mid-year-population-estimates/archive); accessed 29 May 2015)

** The positive value is an additional cost per 5 year old child in the population, whereas the negative values are expected savings per 5 year old child, in comparison with the dental treatments costs in the baseline year 2001/02.

*** Our assumption was that the cost of the toothbrushing programme was constant over time. Hence there is little variation in the average cost per 3–4 year old child in the Scottish population over time.

### Deprivation

**Fig 3:**
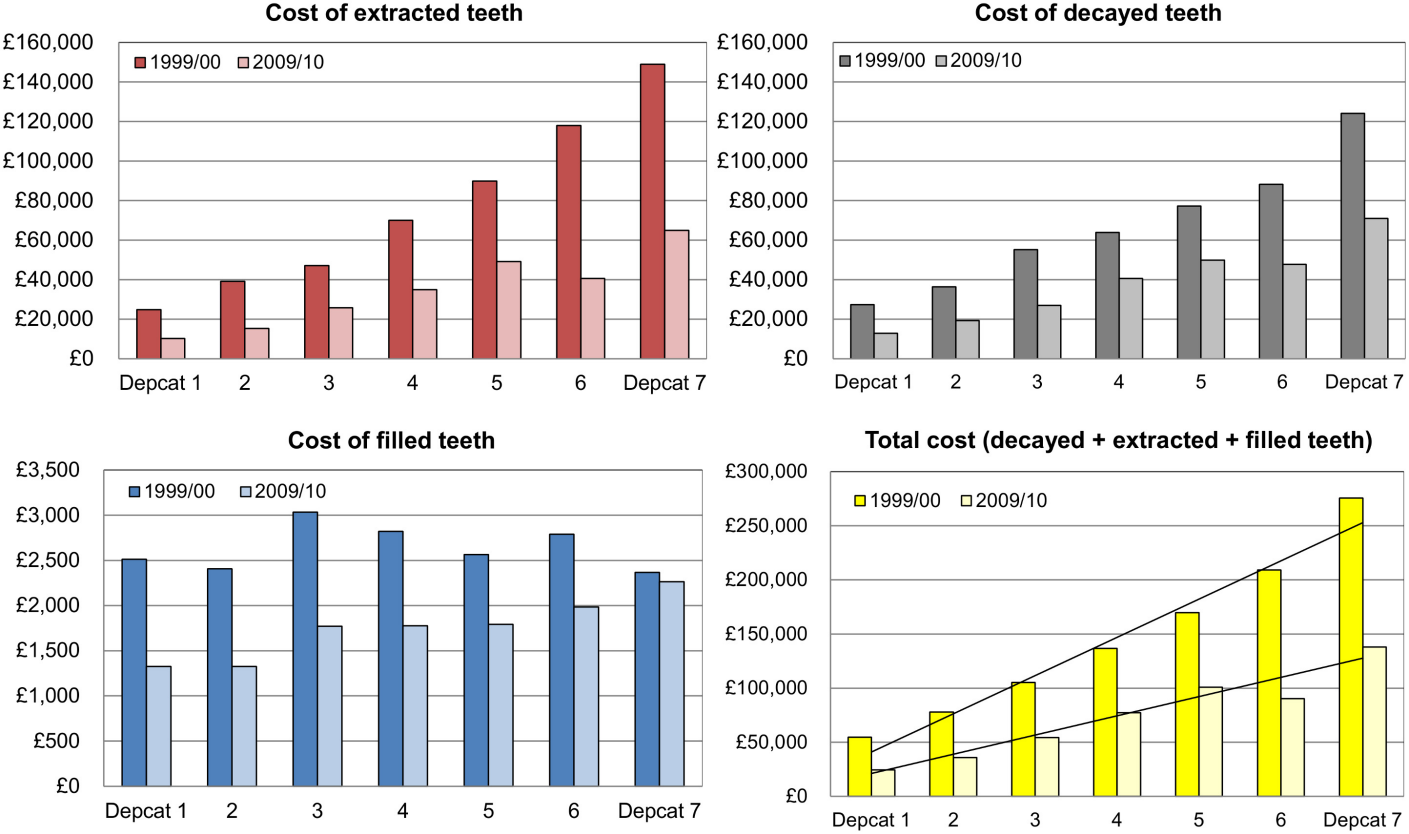
Costs of decayed, extracted and filled teeth per 1000 population, by Depcat (Depcat 1 = least deprived, Depcat 7 = most deprived).

The results of the population standardised analysis per hypothetical cohort of 1000 children per Depcat are shown in Fig 3. The highest expected savings were observed for the most deprived children. The results of the population standardised analysis by deprivation category showed that for the cohort of the most deprived (Depcat 7) the savings resulting from the decrease in the total cost of treatment in primary teeth from 1999/00 to 2009/00 was £137,348 (49.9% of the 1999/00 costs for the most deprived), whereas for the least deprived cohort (Depcat 1) the expected saving was £30,174 (55.3%). For the seven Depcat categories combined the reduction in total costs from 1999/00 to 2009/00 was 49.3% (£507,537). Fig 3 illustrates that the costs of extracted and decayed teeth were much higher in Depcat 7 than in Depcat 1. The gradient in costs of fillings was not prominent. All three cost components analysed (costs of decayed, extracted and filled teeth) decreased over time in each Depcat. The largest decrease, both measured as a percentage of the costs in 1999/00 and in monetary terms, was seen for extracted teeth: for all seven Depcats combined the decrease was £297,124 (55.2%). Substantial decrease in costs was also shown for decayed teeth: £204,155 (43.2%). Although the costs of filled teeth decreased over time, the reduction was not as pronounced: the cumulative decrease across all Depcats was £6,258 (33.8%). It should be noted, that the results of this population standardised analysis by deprivation cannot be compared with the overall Scotland-wide costs / expected savings presented in this paper. This is due to the fact that standardisation was based on hypothetical cohorts of 1000 children per Depcat.

### Sensitivity analysis

**Fig 4:**
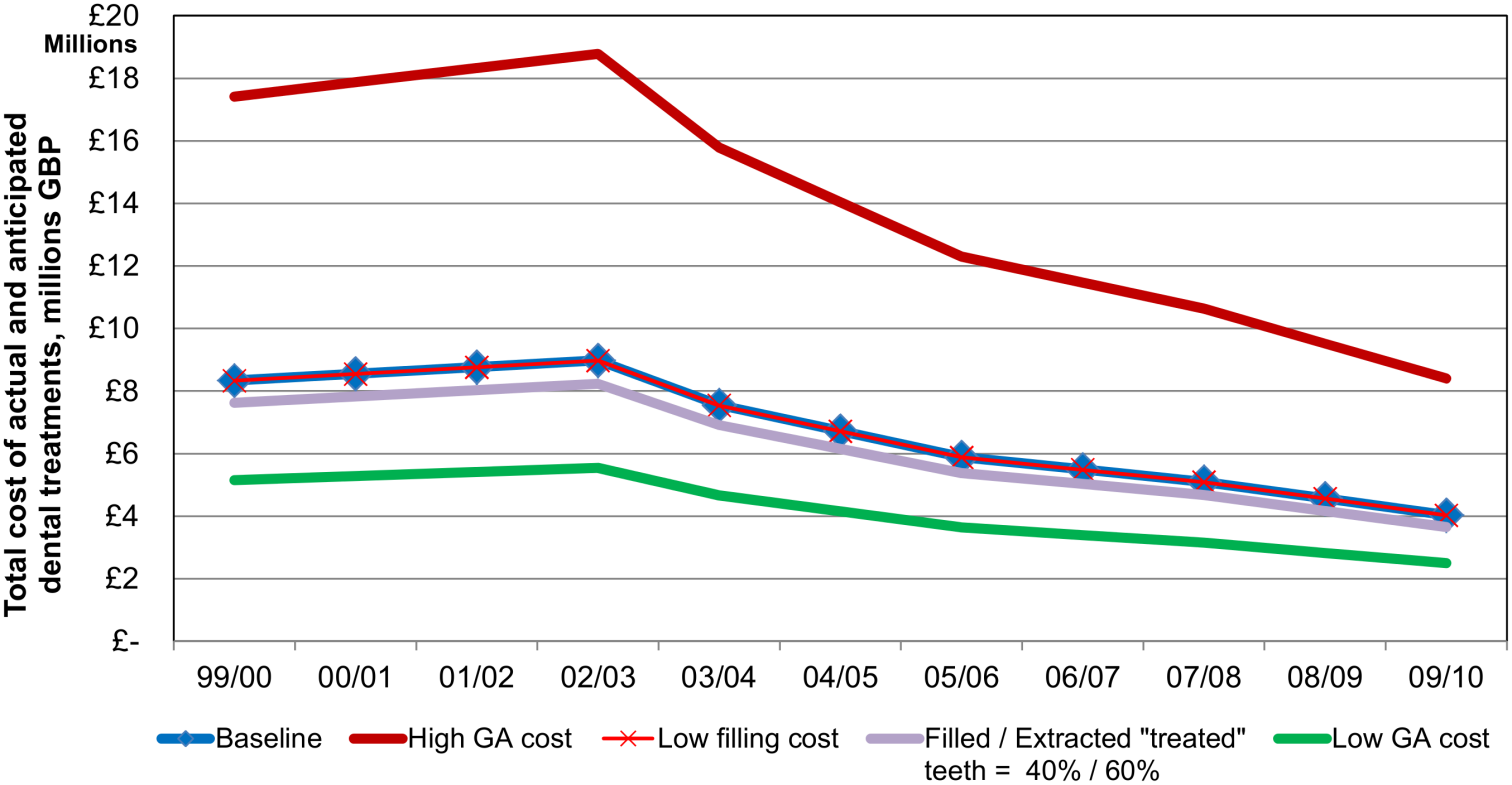
Sensitivity analysis: total cost of actual and anticipated dental treatments—Scotland, 1999/00-2009/10 financial year. Total cost of actual and anticipated dental treatments is a sum of calculated annual costs of extractions, filled teeth and anticipated further cost of decayed teeth.

Sensitivity analysis of the total cost of dental treatments showed that most uncertainty was observed in case of ‘low GA cost’ and ‘high GA cost’ scenarios (Fig 4 and Table 4). For example, in 2001/02 the ‘low GA cost’ scenario total cost of dental treatments was £5,410,531 and in case of ‘high GA cost’ it was £18,325,312. In 2009/10 the costs in these scenarios were £2,501,964 and £8,402,746 respectively.

## Discussion

As discussed in our previous paper [10], the improvement in dental decay levels in Scotland is unlikely to have been part of a secular change. The trend of the mean d_3_mft prior to the start of the nursery toothbrushing programme was flat and then increased over time [10, 34]. The explanation is also unlikely to be due to other sources of fluoride: fluoride supplement use in Scotland is not recommended [35] and fluoride varnish programs did not commence until 2009 apart from a small pilot area [36].

The data on the total implementation costs of the nursery toothbrushing programme collected from the Scottish Health Boards showed great variation in terms of both total costs per Health Board and the cost per child. This can be explained by the fact that there is a considerable degree of variation in the ways the toothbrushing intervention is organised and delivered. This is due to many parameters, such as a varying degree of rurality and remoteness of the areas (e.g., urban areas with high population density, large rural areas with dispersed population or small remote islands areas [37]), population density, various geographical sizes, and slightly different ways of organising the toothbrushing training for the nursery staff, overseeing and supporting the delivery of the programme in nurseries. For example, some of the Health Boards employ oral health assistants, other—oral health support workers, toothbrushing assistants, oral health educators, dental health support workers, or a combination of these. Some of these positions are of different job bands and Health Boards of different sizes employ different numbers of these staff members. Staff travel costs can also vary considerably depending on the size, rurality and remoteness of the Health Board.

The results of this study show that the total estimated costs of actual and anticipated dental treatments decreased over time with the costs in 2009/10 being less than half of those in 2001/02, when the nursery toothbrushing program was rolled-out across Scotland. The major components of the total dental treatments cost were the cost of extractions and the anticipated further cost of decayed teeth, as both of these costs were influenced by a high cost of dental extraction under GA. The sensitivity analysis indicated that the ‘high’ and ‘low’ GA cost scenarios showed most uncertainty in relation to the amount of expected savings.

The range of costs of a GA procedure used in this study (i.e. £653.25 in the main cost of tooth extraction calculation, and £393.22 and £1,393.89 in the sensitivity analysis) is comparable with the costs of a dental GA procedure cited in other sources. Namely, in a report by the Personal Social Services Research Unit (PSSRU), Great Britain, a child’s inpatient stay for dental extraction cost £1,146 [38]; a paper comparing dental GA costs per child with the costs of a dental sedation procedure cited hospital based GA costs in the range £195-£616 in 2003 GBP [39]; and in a costing report by the NHS National Institute for Health and Care Excellence the GA costs were £229 and £720 per child [40].

The population standardised analysis for the hypothetical cohort of 1000 children per Depcat showed that the largest decrease in modelled costs was for the most deprived cohort of children. In 2009/10 the overall cost of treatment in primary teeth in the Depcat 7 cohort was half of the cost in 1999/00 (Fig 3). A successful reduction in the social gradient was demonstrated (in addition, a detailed analysis of improvement in child dental health by deprivation category was reported in our previous paper [10]). One could hypothesise that the nursery toothbrushing programme should most affect the more deprived children due to the high baseline dental decay levels in these children, and also because prior to the intervention home toothbrushing was more likely to be carried out on a regular basis within the less deprived families [41, 42].

The economic evaluations of caries prevention interventions published to date were in regard to randomised controlled trials, small to medium sized cohort studies [43–45] or modelling using hypothetical populations [14]. Moreover, in the above mentioned studies toothbrushing was usually one of the components of a complex caries preventive program, rather than a standalone intervention. The authors were not able to find any previously published papers that evaluated the costs and/or cost savings of an extensive nursery or school toothbrushing program. The only two economic analyses found, which related to the use of fluoride toothpaste, were a cost-effectiveness analysis of a postal toothpaste programme in the North West of England [16] and a cost-benefit analysis of an oral health promotion project using fluoride toothpaste in Nepal [17]. The costs reported in these two studies, however, are not directly comparable with the costs of our study. The Davies et al. study involved 6,781 children aged 12 months at the baseline and their intervention was a postal distribution of toothpaste four times a year, hence not comparable to providing a nation-wide daily supervised toothbrushing program in nurseries. Yee et al. evaluated a broader advocacy project to increase the availability and consumption of fluoride toothpaste in the age group 6–18 years and their study took place in a developing country, also not directly comparable with the context of this study. Moreover Yee et al. investigated projected caries levels in the permanent dentition, whereas our research analysed historical data on primary teeth caries.

Davies et al. found that the cost of the four-year postal programme per child was £27.93. A previous paper by the same first author [46] indicated that the children invited to participate in the trial were born in 1993–1994. Thus we took 1995 as the cost year for the costs stated in the study. Inflated at a 3% per year rate to our toothbrushing costs data collection year of 2011 this equals £44.82, which means an annual cost of approximately £11.20 (which is £44.82 divided by 4 years). For comparison, the average annual cost per 3–4 year old child in the population in our study in 2011/12 was £15.82 with the range by Health Board from £4.76 to £76.88. The highest costs per capita were observed for island Health Boards, such as Orkney, Shetland and Western Isles, due to remoteness, low population density, higher ratio of NHS staff delivering and overseeing the programme to the number of 3–4 year old children in the Health Board, and NHS staff travel costs. According to Davies et al., at the end of their study period the difference between mean d_3_mft of children in the control (‘do nothing’) group and that of the intervention group was 0.42 (2.57 vs. 2.15). Over the timeframe of our study, the mean d_3_mft in five-year-olds decreased by 1.03 (from 2.55 in 1999/00 to 1.52 in 2009/10).

### Limitations

Cost savings were calculated and used as a proxy to represent the opportunity cost associated with freed-up resources due to improved dental health in the absence of data mechanisms to track the alternative use of freed-up appointments. These cost savings and associated health gains however are an underestimate of the full benefit of the programme. Besides the dental health improvements of children receiving the toothbrushing there are additional health benefits arising from dental care received in the now freed-up appointment slots hence future studies could attempt to capture these data.

A further limitation of this study was the assumption that the cost of the nursery toothbrushing program was constant over time. In reality, the nursery toothbrushing was rolled out Scotland-wide in 2001 and by 2005/06 the average nursery participation rate reached around 80%, further increasing to 95% in 2006/07, and staying at or above this level onwards. Nonetheless, taking in account start-up costs of engaging nurseries to participate in the programme and monitoring-related work in the early years on the programme, we believe that our assumption of a constant cost is accurate. Another limitation was that in the absence of reliable historical data on the numbers of zero to five year old children that had their teeth extracted under GA, we used the numbers of missing teeth per child from the national dental inspections and the assumptions that if a child had one tooth missing it was extracted under LA, and if two or more teeth were missing it was done under GA. The authors made an attempt to investigate GA data from hospital discharge database (provided on request by ISD), however, substantial problems with data quality were discovered, especially for the earlier historical periods [31], mainly due to under-reporting.

## Conclusions

NHS costs that were associated with actual and anticipated dental treatments for five-year-old children decreased dramatically over time. The findings, based on our assumptions, suggest that within three years the expected cost savings (freed-up resources) outweighed the costs of implementing the toothbrushing program and by eight years the expected cost savings were in excess of two and a half times these costs. These expected resource savings were associated with the national roll-out of the nursery toothbrushing program and an improvement in children’s oral health. In economic terms the toothbrushing program therefore represents an example of a preventative spend and a ‘win win’ scenario of both reduced costs and health gains in child oral health outcomes. A population standardised analysis of hypothetical cohorts of 1000 children per Depcat showed that the largest decrease in costs and associated dental health gain occurred in the cohorts of children within the highest deprivation categories. Thus, a successful reduction across social gradient was demonstrated revealing that the nursery toothbrushing programme not only reduces costs and improves dental health but also reduces health inequalities. Using any decision making criteria for optimal allocation of resources this programme appears to be highly worthwhile.
